# Enhancing Reliability and Safety in Industrial Applications: Assessing the Applicability of Energy *b*-Value to Composites

**DOI:** 10.3390/ma17020447

**Published:** 2024-01-17

**Authors:** Doyun Jung, Jeonghan Lee

**Affiliations:** Korea Atomic Energy Research Institute, 111, Daedeok-Daero 989-gil, Yusenong-gu, Daejeon 34057, Republic of Korea

**Keywords:** structural integrity, fracture behavior, AE testing, GFRP

## Abstract

This study investigates the fracture behavior of glass fiber-reinforced plastic (GFRP) under various loading conditions using acoustic emission (AE) testing. Using fracture tests and time series analysis of AE signals, parameters such as *b*-value, improved *b*-value (I*b*-value), and energy *b*-value (*b*_e_-value) were examined to understand crack initiation, growth, and structural failure. The stress–strain curve revealed distinct responses during tensile and step loading, and time series analysis highlighted variations in amplitude, AE energy, and Kaiser and Felicity effects. Under tensile loading, the I*b*-value exhibited a linear decrease, while step loading introduced complexities, including the Felicity effect. The *b*_e_-value, incorporating energy considerations, fluctuated, providing insights into micro-cracks and macro-cracks. Statistical analysis demonstrated a consistent decrease in the *b*_e_-value, emphasizing its potential for long-term monitoring. This study provides a comprehensive technique for assessing composite material fracture behavior, enhancing understanding for critical applications in hydrogen storage vessels and pressure pipes as well as advancing reliability and safety in industrial sectors.

## 1. Introduction

Non-destructive testing (NDT) is a technique employed for the inspection and assessment of structures to detect damage without causing harm. This method involves evaluating the condition of an object without resorting to destructive means. Various NDT techniques, including ultrasonic testing [1,2], magnetic particle inspection [3,4], and X-ray testing [5], find application in industrial fields, such as turbine generators [6,7], rotating equipment [8], and pressure piping lines [9]. However, recent attention has shifted towards AE testing [10] driven by its ease of real-time monitoring and surveillance. AE testing is a method that analyzes acoustic waves resulting from rapid stress changes. Elastic waves generated by internal cracks in the material propagate through the material and are detected by an AE sensor, a device that converts vibrations into electrical signals. During this process, some of the acoustic waves may be released into the air and could be heard as a breaking sound. AE testing stands out for its passive monitoring approach, where recorded AE signals offer information related to damage, encompassing aspects like cracks [11], leaks [12], and friction [13]. This information is meticulously captured as a time series, providing a detailed chronicle of changes in damage from the initial installation to the eventual dismantling of the structure.

The relationship between AE signals and external loading relies on the Kaiser effect [14] and Felicity effect [15]. The Kaiser effect demonstrates elastic behavior during reloading, ceasing only upon reaching the previous maximum stress level. In contrast, the Felicity effect is characterized by significant acoustic emissions during cyclic loading, even at stress levels below the previously applied maximum. These effects are fundamental principles used to monitor and evaluate internal damage within a structure. The Kaiser effect relies on the damage history at the point of maximum stress, while the Felicity effect uses acoustic signals during cyclic loading. Analyzing recorded AE signals through statistical techniques enables real-time assessment of the current state of damage within the structure. For these reasons, AE testing is applied to pressure vessels, pipelines, and structures to assess real-time structural integrity [16,17,18,19]. The key parameter for analyzing structural integrity through AE testing is the *b*-value, which detects damage (cracks) in structures. The concept of the *b*-value originated from seismological research, initially proposed by Gutenberg and Richter in 1944 [20]. According to this law, smaller earthquakes are observed to occur with significantly greater frequency than larger ones. This relationship is typically depicted in a log-linear format (See Equation (1), Section 2.3), effectively demonstrating that the occurrence rate of earthquakes decreases exponentially as their magnitude increases [20]. For this reason, this law has become a significant research topic in the field of damage assessment for engineering structures [10].

Over the past decades, the *b*-value has been widely employed to predict and analyze damage in rocks [21] and concrete [22]. This analysis utilizes amplitude as a direct indicator of damage, with a decrease in the *b*-value corresponding to crack growth. Researchers conducting fracture tests using small-scale samples have investigated the characteristics of the *b*-value to clarify the precursor to damage and failure in solid materials. Scholz et al. [23] associated the *b*-value with the stress state of rocks and found that the *b*-value increases at stress levels below 90% but starts to decrease beyond that point. Shiotani et al. [24,25]. Conducted AE research on concrete specimens under bending and cyclic loading conditions, discovering that microcracks generate a large number of events with small amplitudes (increase in *b*-value), while macrocracks produce fewer events with larger amplitudes (decrease in *b*-value). Ohno et al. [26] pointed out that tensile cracks generate high amplitudes, whereas shear cracks produce smaller amplitudes. The application of the *b*-value has been successfully demonstrated in the evaluation of cracks in reinforced concrete beams under various loading conditions. Shiotani [25] and collaborators [21] introduced the concept of the improved *b*-value (I*b*-value) by integrating statistical values from amplitude distribution analysis. Sagasta et al. proposed the use of the energy *b*-value (*b*_e_-value) [27,28], utilizing the actual energy of AE signals instead of the traditionally used peak amplitude for *b*-value calculations.

Lightweight and high-strength composite materials, such as fiber-reinforced plastics (FRP), are increasingly used in aircraft and spacecraft components due to their advantageous properties [29,30,31]. However, during their manufacturing process, machining operations, like drilling, are necessary, which can lead to a significant issue, namely, delamination [32]. This is most often observed on the exit side near the hole edge, resulting from the drilling process. Delamination can adversely affect the support strength of the joint, reduce the service life, and compromise the safety of the structure. In response to these challenges, various solutions, such as sandwich panels and honeycomb structures, have been developed, but failures due to delamination still occur [33]. To address this, research is being conducted on the application of the *b*-value for evaluating the integrity and in-service soundness of these composite materials. In FRP, the *b*-value, a parameter used to assess material integrity, changes in response to the type of cracks present; it increases when microcracks are dominant and decreases when macrocracks become dominant. The I*b*-value (an indicator of crack progression) is observed to consistently decrease from the initiation of a crack to the final failure of the material [34,35,36]. This behavior in FRP is similar to what has been observed in other materials, such as rocks and concrete. Despite the potential of the *b*-value in evaluating material integrity, there is currently no study that focuses specifically on evaluating the fracture behavior of FRP using the *b*-value. This gap highlights the need for further research in this area to better understand and mitigate the risks associated with delamination in composite materials used in critical applications, like aircraft and spacecraft.

**Figure 1:**
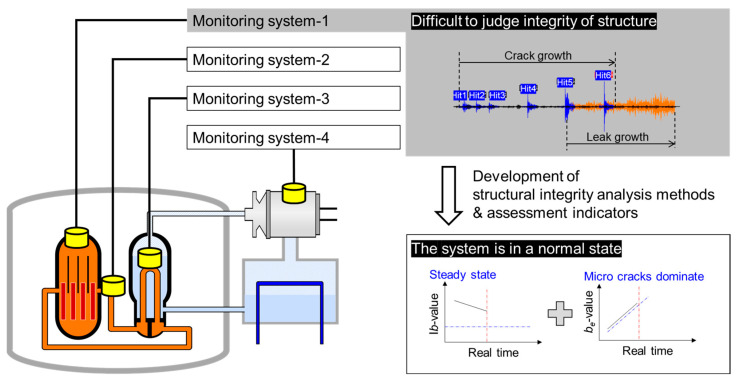
Summary of final research objectives.

In this study, we fabricated test specimens made of woven GFRP, which is one of the most widely used materials in the industry, featuring a hole in the center. To discuss the applicability of the *b*_e_-value, we progressively increased the load on the specimens until failure while acquiring AE signals. We utilized mechanical parameters, such as the I*b*-value, stress, and strain (life), and evaluated the applicability of the *b*_e_-value by comparing it with the already verified I*b*-value. This research presents a tailored structural integrity assessment method for composite material structures, such as hydrogen storage vessels and pressure pipes. We identified descriptors that are easily interpretable by operators of monitoring systems, demonstrating their practicality and potential for application in various fields. Additionally, this study suggests that these descriptors can be applied not only to composite materials but also to a wide range of engineering structures (see Figure 1). If the *b*-value, I*b*-value, and *b*_e_-value are successfully demonstrated in composite materials, these indicators could potentially be used not only for assessing the integrity of composite material structures but also for evaluating the location, timing, severity, and expected lifespan of phenomena, such as delamination occurring during drilling. Future research should prioritize the refinement of assessment metrics and the enhancement of reliability in methods used for evaluating cracks in composite materials. This effort should involve extensive testing using a large number of specimens, which would provide a more robust and comprehensive understanding of the behavior and properties of these materials under various conditions. This signifies that these indicators hold potential for extension and application in a broader range of research and application programs.

## 2. Experimental Procedures

### 2.1. Specimens

**Figure 2:**
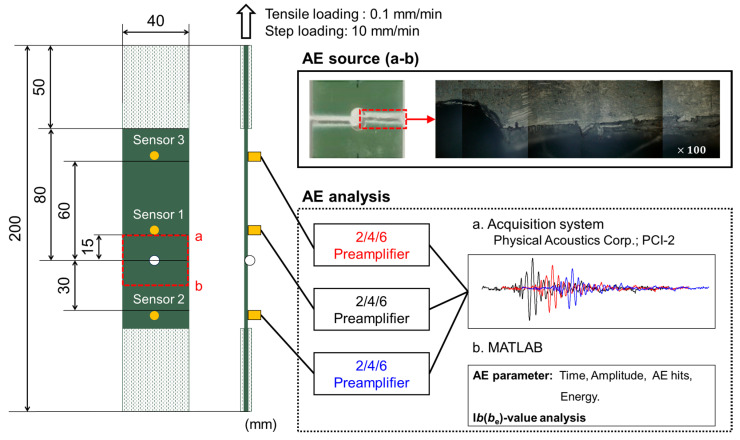
Test specimen and AE sensor.

A GFRP sheet, 2 mm thick and consisting of glass fiber and epoxy resin from Murakami Dengyo Co., Ltd., Yokohama, Japan, was employed in the experiments (see Figure 2). The glass fibers were arranged in a plain-woven fabric configuration, constituting 52.3 vol% of the specimen. Woven GFRP plate specimens, designed in accordance with ASTM D3039 [37] standards and featuring a center hole, were utilized for mechanical testing. To prevent damage from the test jig, GFRP tabs were affixed to each end of the specimen. Each specimen underwent meticulous preparation, involving the creation of a 2 mm diameter hole at the center using a drill specifically designed for composite materials. The tensile test, which was conducted in accordance with the guidelines described in ASTM D3039, was performed in a universal testing machine until failure at a loading rate of 0.1 mm/min. In step loading, the loading/unloading speed is 10 mm/min. In the tests involving both cyclic and tensile loading, a single woven GFRP specimen was utilized for each type of load.

### 2.2. Acoustic Emission Testing

AE signals were recorded using a digitizer (Physical Acoustics Corp.; PCI-2, Princeton Junction, NJ, USA) with a per-channel sampling rate of 10 MHz during each test. The tests utilized 2/4/6 preamplifiers (Physical Acoustics Corp., Princeton Junction, NJ, USA) with a 40 dB_AE_ gain. For AE testing, a PICO sensor from Physical Acoustics Corporation (PAC; Princeton Junction, NJ, USA), operating in the frequency range of 200 to 750 kHz, was chosen. Three AE sensors, strategically positioned at various distances from the hole, were affixed to each specimen using silicon grease (HIVAC-G, Tokyo, Japan) and secured with vinyl tape (refer to Figure 2). Specimens experienced fractures near the center hole due to stress concentration. Table 1 summarizes the test conditions based on previous research for AE monitoring. Guard sensors were applied at both ends of the specimen, and AE signals exceeding 40 dB_AE_ as measured by the sensors were recorded, thereby filtering out background noise.

### 2.3. b-Value Analysis

**Figure 3:**
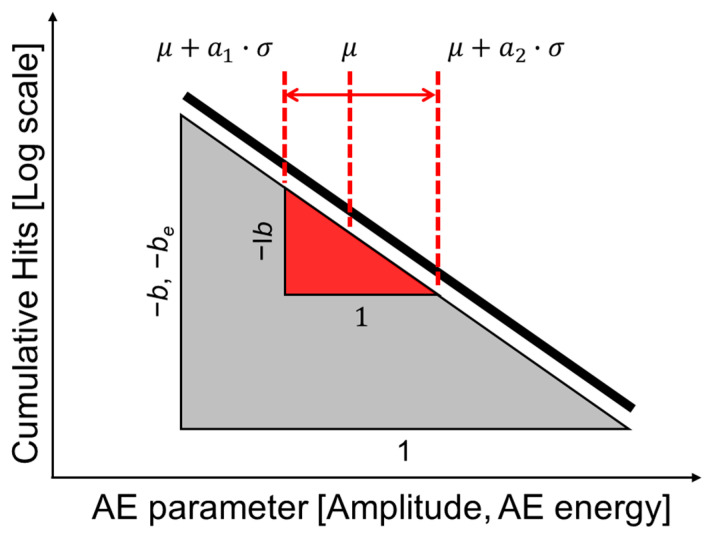
The definitions of *b*-value, I*b*-value, and *b*_e_-value.

The *b*-value analysis (see Figure 3), which was originally developed by Gutenberg [20] for seismological applications, is expressed by the formula:

(1) $$\log_{10}\left(N\right)=a-b\times A$$

where *A* is the amplitude of AE signal in decibels (dB_AE_), *N* is the total number of AE hits, *a* is an empirical constant, and *b* is the slope of the linear relationship. This analysis utilizes amplitude as a direct indicator of damage, with a decrease in the *b*-value corresponding to crack growth. For enhanced analysis, the I*b*-value [24] was introduced, taking into account specific statistical parameters of the amplitude distribution. The I*b*-value is calculated using the mean (*μ*), standard deviation (*σ*), lower amplitude (*μ* − *a*_1_ × *σ*), and upper amplitude (*μ* + *a*_2_ × *σ*) values:

(2) $$Ib=\frac{\log_{10}N\left(\mu -a_{1}\cdot \sigma \right)-\log_{10}N\left(\mu +a_{2}\cdot \sigma \right)}{\left(a_{1}+a_{2}\right)\cdot \sigma }$$

In the field of AE, often considered as micro-seismology, the *b*(I*b*)-value is extensively used to assess fracture processes. Similar to its application in the engineering field, the *b*-value in AE is associated with the number of events and the amplitude of those events. High *b*-values are connected to numerous small-amplitude AE events, such as those from micro-crack formation and slow crack growth. Conversely, low *b*-values indicate faster-growing or unstable crack formation. The *b*-value systematically changes during different stages of the fracture process, making it a valuable indicator for estimating failure development. In this study, following the commonly accepted approach, it was assumed that *α*_1_ = *α*_2_ = 3. Sagasta et al. [28] proposed the energy *b*-value (*b*_e_-value) as follows:

(3) $$\log_{10}N\left({\mathrm{AE}}_{e}\right)=a-b_{e}\times \log_{10}{\mathrm{AE}}_{e}$$

The proposed energy *b*-value (*b*_e_-value) for assessing local damage in reinforced concrete structures under dynamic loads, based on the traditional *b*-value, utilizes the true energy of AE signals instead of the peak amplitude. Experimental application demonstrates its potential for damage assessment, with the *b*_e_-value responding when severe damage occurs in critical areas of the structure.

## 3. Results and Discussion

**Figure 4:**
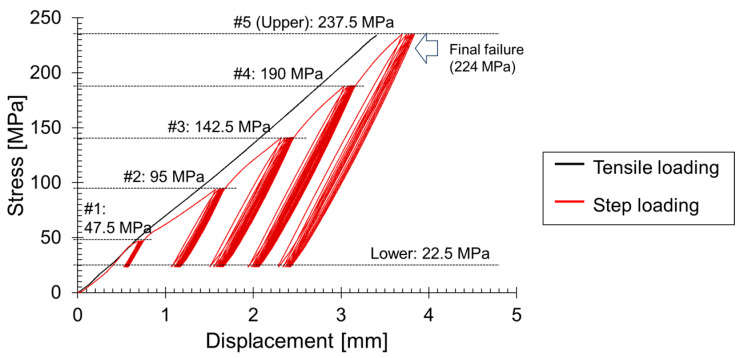
Stress–strain curve from GFRP fracture test.

Figure 4 presents the stress–strain curve obtained from a fracture test using the GFRP specimen with a center hole (refer to Figure 2). The black line illustrates the results of the tensile fracture test, with the maximum stress (237.5 MPa) divided into five increments for step loading. The lower limit value in this case is 22.5 MPa, and all conditions are summarized in Figure 2. To distinguish AE signals generated at each stage, a step loading test progressed from #1 to #2. After repeating #1 from 22.5 MPa to 47.5 MPa ten times, the first stage (#1) concluded upon reaching 22.5 MPa, and then #2 (95 MPa) began. Throughout tests from #1 to #5, the test specimen remained continuously attached to the universal testing machine, maintaining independence from external forces.

**Figure 5:**
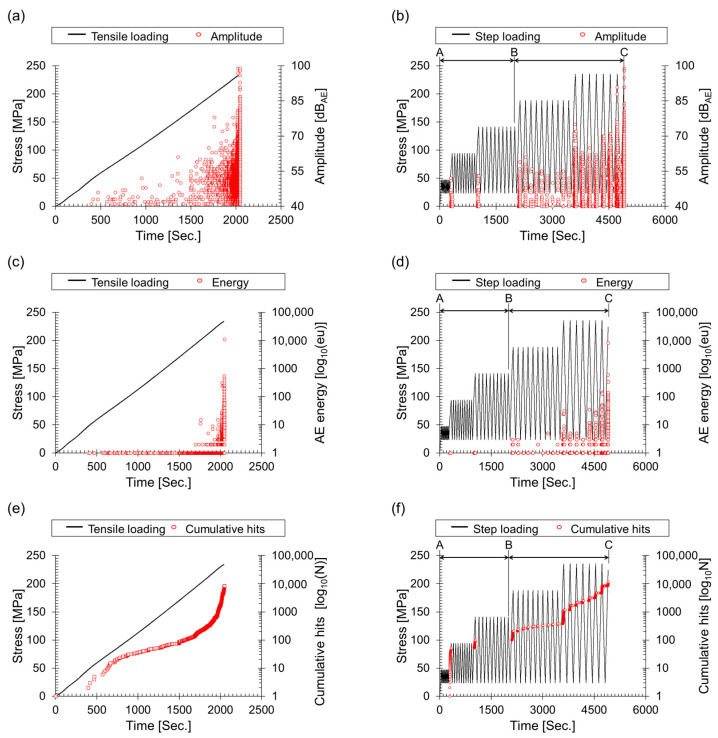
Analysis of parameters under tensile and cyclic loading: (**a**) amplitude during tensile loading, (**b**) amplitude during cyclic loading, (**c**) AE energy during tensile loading, (**d**) AE energy during cyclic loading, (**e**) cumulative hits during tensile loading, (**f**) cumulative hits during cyclic loading.

Figure 5 illustrates the variation of AE parameters concerning stress. Time series analysis was employed to compare and analyze AE signals, I*b*-value, and *b*_e_-value based on the fracture test results.

The maximum amplitude consistently increased under both loading conditions (Figure 5a,b). The Kaiser effect occurs in the elastic region of composite materials, while the Felicity effect is recognized in the plastic deformation region. The A–B segment in Figure 5b,d,f represents the Kaiser effect region, while the B–C segment indicates the Felicity effect region. The Felicity effect generates more AE hits during step loading than tensile loading because it produces signals even during unloading.

Figure 5c,d illustrate the AE energy behavior in response to load changes. During the AE test, all measured signals were converted to AE energy and displayed on a logarithmic scale for clarity. AE energy shows a wide dispersion in the yielding region compared to amplitude, consistent with previous reports and highlighting the correlation between AE signal energy and yielding deformation. during tensile loading, 8460 AE signals were generated, while 11,599 signals were recorded during step loading (Figure 5e,f).

Theoretically, AE signals are generated from the initiation, propagation, and fracture of cracks. Consequently, AE hits and the *b*-value serve as crucial indicators for evaluating the integrity of materials. Within this framework, the rate of AE hits is utilized as a metric to gauge the degree of crack activity. Nonetheless, this metric encounters limitations in differentiating between scenarios dominated by micro-cracks and those where crack propagation has ceased. In micro-crack-dominated scenarios, an elevated rate of AE hits is typically observed, whereas in contexts characterized by macro-cracks or halted crack growth, a notable decrease in the AE hit rate is evident. Such ambiguities can result in significant inaccuracies when assessing structural integrity.

The *b*-value, which evaluates structural soundness, is determined by plotting the amplitude of AE hits on a frequency distribution chart and analyzing the resultant slope. In this methodology, the amplitude serves as an indicative parameter for crack size, with the *b*-value escalating in the presence of micro-cracks and diminishing in macro-crack-dominant scenarios. In contrast, the I*b*-value is derived using statistical techniques to identify representative segments for crack evaluation. This approach, which is relatively static, leverages current value computations, as opposed to the *b*-value’s dynamic, trend-based evaluative method.

**Figure 6:**
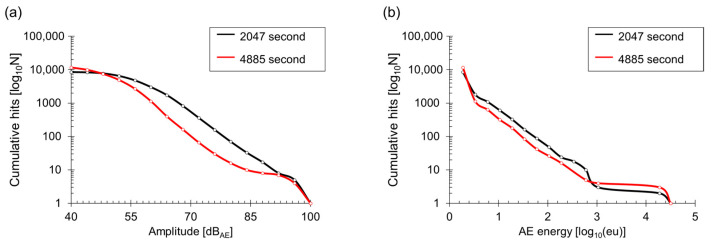
Frequency distribution of amplitude (**a**) and AE energy (**b**).

To calculate the I*b*(*b*_e_)-value, a frequency distribution is necessary, requiring a determination of the class. During the fracture test, the amplitude and AE energy of all recorded signals were displayed in a frequency distribution. Figure 6a represents the variance curve of amplitude divided into 16 intervals based on past research experience. AE energy was partitioned into 16 intervals based on maximum and minimum values (Figure 6b). The overall slope in Figure 6a corresponds to the previously mentioned *b*(I*b*)-value method that evaluates the slope of specific intervals to assess the cracks in the structure. The slope of the frequency distribution was calculated using the least squares method each time AE hits accumulated to 5.

**Figure 7:**
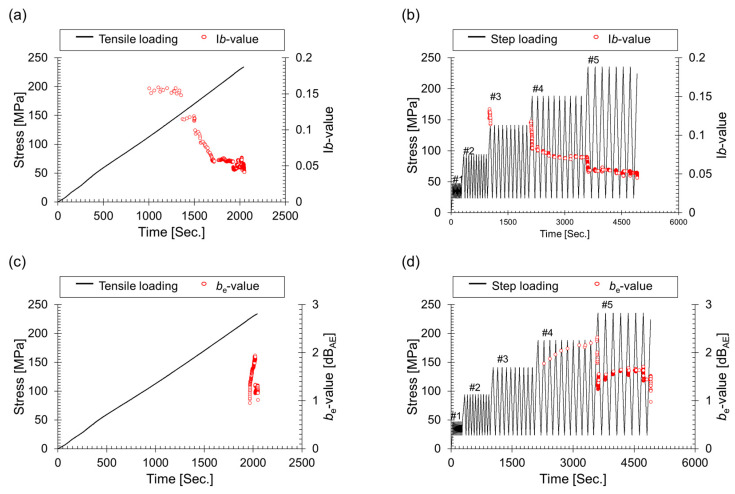
Fluctuations in I*b*(*b*_e_)-values under different loading conditions: I*b*-value during (**a**) tensile loading and (**b**) cyclic loading. Similarly, *b*_e_-value during (**c**) tensile loading and (**d**) cyclic loading.

In Figure 7, fluctuations in I*b*(*b*_e_)-values are observed under different loading conditions. During tensile loading, the I*b*-value consistently decreases linearly from crack initiation to final failure, aligning with similar observations in reports from the concrete and composite material fields. Composite materials, renowned for their versatility, exhibit a range of failure modes that significantly contribute to their structural behavior. One particularly noteworthy phenomenon is the manifestation of matrix cracking, prominently observed immediately preceding ultimate failure. This distinctive characteristic introduces a discernible scatter in the I*b*-values, a trend perceptible from the 1800 s mark in Figure 7b. Indeed, this process closely resembles the analysis of acoustic emission (AE) signals in mechanical systems comprising diverse combinations of components and materials. The distinct variations in I*b*-values across five loading conditions highlight the material’s nuanced response to different stress scenarios. Notably, the most significant reduction in *b*-value is evident during the initial step of #4, indicating pronounced crack growth and emission at a higher load (#4) compared to the preceding load (#3). Further insights into crack growth and emission under increased loads are revealed by specific instances, such as the substantial drop in *b*-value during the initial step of #4. Additionally, the application of the Felicity effect from #3 onwards contributes to observed variations in I*b*-values throughout the steps of #3. In #2, the presence of the Kaiser effect region is apparent, with I*b*-values changing only during the first cycle. The GFRP subjected to step loading experiences failure in the 8th cycle of #5, exhibiting an I*b*-value of 0.4, which is identical to the tensile loading value. Notably, certain studies assign significance to values through repetitive experiments, using them as critical criteria for evaluating the operational durability of structures.

Figure 7c presents the *b*_e_-value categorized into increasing and decreasing segments. The conventional variation of *b*-values can be attributed to micro-cracks and macro-cracks. Micro-cracks, occurring predominantly at the crack tip, result in the generation of numerous AE signals with small AE energy. Hence, an accumulation of signals in the low-energy range leads to an increase in the *b*_e_-value. On the other hand, in the high-stress regime, there is the initiation of macro-cracks that penetrate through the micro-cracks. Macro-cracks accumulate in the high AE energy range of the frequency distribution, causing an increase in the *b*_e_-value. The trend of increases and decreases in *b*_e_-value can be used to assess the condition of cracks in the structure.

In practical industrial settings, the demand for static indicators is paramount to ensure easy interpretation and seamless application. To meet this demand, we adopted a method that harmonizes insights from Equation (2) to Equation (3), aligning with the observed trend in step loading. This unified approach facilitates a more static assessment of crack conditions, catering to the pragmatic needs of real-world applications in industrial contexts.

In our methodology, representative intervals on the AE energy derivative curve were judiciously selected, leveraging statistical parameters, such as the mean and standard deviation. The subsequent calculation of the slope within these carefully chosen intervals provides a practical and actionable indicator for assessing crack conditions. The assumption of *α*_1_ = *α*_2_ = 3, mirroring the approach used for the I*b*-value, ensures consistency in the application of statistical parameters.

The results obtained for the *b*_e_-value, which is akin to the I*b*-value, consistently displayed a decreasing trend, transitioning from 4 to 1 under tensile-loading conditions. This consistency underscores the reliability and stability of our methodology in providing valuable insights into crack conditions. By seamlessly integrating dynamic insights derived from the continuous changes in the *b*_e_-value with the stability offered by static indicators, our unified approach effectively bridges the gap between comprehensive understanding and practical utility in industrial contexts. This method holds promise for efficient crack condition assessment and monitoring in GFRP, contributing significantly to enhanced structural health management across diverse industrial applications.

## 4. Conclusions

In this study, we conducted a comprehensive investigation of the fracture behavior of GFRP under different loading conditions, utilizing AE testing and analyzing parameters, such as I*b*-value, and *b*_e_-value. The experimental procedures involved fracture tests on GFRP specimens, capturing AE signals through time series analysis. Our findings provide valuable insights into the structural integrity assessment of composite materials, particularly relevant for applications such as hydrogen storage vessels and pressure pipes.

The stress-strain curve derived from the fracture test illuminated the material’s behavior under varying loads, with distinctive responses observed during tensile and step loading. The time series analysis of AE parameters revealed nuanced variations in amplitude, AE energy, and the occurrence of Kaiser and Felicity effects. The I*b*(*b*_e_)-value analysis, considering statistical parameters, offered a detailed assessment of crack initiation, growth, and structural failure.

Under tensile loading, the I*b*-value exhibited a linear decrease from crack initiation to final failure, consistent with observations in concrete and composite material studies. The step-loading conditions introduced complexities, including the Felicity effect, resulting in distinct variations in I*b*-values. The *b*_e_-value, incorporating energy considerations, showcased fluctuations attributed to micro-cracks and macro-cracks, providing further insights into the evolving nature of structural damage.

The application of statistical analysis to the *b*_e_-value, with assumptions similar to the I*b*-value, revealed a consistent decrease, emphasizing the potential of these parameters for long-term monitoring. The improved *b*_e_-value demonstrated a continuous decline, aligning with both tensile and step loading scenarios.

In conclusion, this study describes a comprehensive technique for assessing the fracture behavior and structural integrity of composite materials. The detailed analysis of AE parameters, especially the I*b*(*b*_e_)-value, offers a valuable tool for real-time monitoring and long-term evaluation, enhancing our understanding of the operational principles of composite structures in critical applications. These insights are crucial for advancing the reliability and safety of composite material-based structures in various industrial sectors.

## Figures and Tables

**Table 1:** Test Conditions for AE Monitoring.

Threshold		Amplifier	Analog Filter		Sampling Condition		
Type	dB_AE_	dB_AE_	Lower	Upper	Rate	Pre-Trigger	Length
Fixed	35	40	1 kHz	1 MHz	10 MHz	50 μs	1k μs

Length: recording time for each acoustic emission (AE) signal after the AE signal exceeded the threshold value.

## Data Availability

Data are contained within the article.

## References

[1] Rakotonarivo S.T., Payan C., Moysan J., Hochard C. (2018). Local Damage Evaluation of a Laminate Composite Plate Using Ultrasonic Birefringence of Shear Wave. Compos. B Eng..

[2] Wang X., Wang E., Liu X. (2019). Damage Characterization of Concrete under Multi-Step Loading by Integrated Ultrasonic and Acoustic Emission Techniques. Constr. Build. Mater..

[3] Zolfaghari A., Zolfaghari A., Kolahan F. (2018). Reliability and Sensitivity of Magnetic Particle Nondestructive Testing in Detecting the Surface Cracks of Welded Components. Nondestruct. Test. Eval..

[4] Wu Q., Qin X., Dong K., Shi A., Hu Z. (2023). A Learning-Based Crack Defect Detection and 3D Localization Framework for Automated Fluorescent Magnetic Particle Inspection. Expert Syst. Appl..

[5] Dong X., Taylor C.J., Cootes T.F. (2020). A Random Forest-Based Automatic Inspection System for Aerospace Welds in X-ray Images. IEEE Trans. Autom. Sci. Eng..

[6] Mevissen F., Meo M. (2019). A Review of NDT/Structural Health Monitoring Techniques for Hot Gas Components in Gas Turbines. Sensors.

[7] Cacciola M., Pellicano D., Megali G., Calcagno S., Morabito F.C. (2010). Rotating Electromagnetic Field for NDT Inspections. Prog. Electromagn. Res. B.

[8] Drewry M.A., Georgiou G.A. (2007). A Review of NDT Techniques for Wind Turbines. Insight-Non-Destr. Test. Cond. Monit..

[9] Zhou W., Wang J., Pan Z., Liu J., Ma L., Zhou J., Su Y. (2022). Review on Optimization Design, Failure Analysis and Non-Destructive Testing of Composite Hydrogen Storage Vessel. Int. J. Hydrog. Energy.

[10] Mizutani Y., Enoki M., Inaba H., Nakamura H., Nakano M., Shigeishi M., Tomoki S., Shinichi T. (2016). Practical Acoustic Emission Testing.

[11] Du K., Li X., Tao M., Wang S. (2020). Experimental Study on Acoustic Emission (AE) Characteristics and Crack Classification during Rock Fracture in Several Basic Lab Tests. Int. J. Rock Mech. Min. Sci..

[12] Quy T.B., Kim J.-M. (2021). Real-Time Leak Detection for a Gas Pipeline Using Ak-NN Classifier and Hybrid AE Features. Sensors.

[13] Fan Y., Gu F., Ball A. (2010). Modelling Acoustic Emissions Generated by Sliding Friction. Wear.

[14] Kaiser J. (1950). Untersuchungen Über das Auftreten von Geräuschen Beim Zugversuch. Ph.D. Thesis.

[15] Fowler T.J. (1979). Acoustic Emission of Fiber Reinforced Plastics. J. Tech. Counc. ASCE.

[16] Kharrat M., Placet V., Ramasso E., Boubakar M.L. (2016). Influence of Damage Accumulation under Fatigue Loading on the AE-Based Health Assessment of Composite Materials: Wave Distortion and AE-Features Evolution as a Function of Damage Level. Compos. Part A Appl. Sci. Manuf..

[17] Woo S.C., Kim T.W. (2016). High Strain-Rate Failure in Carbon/Kevlar Hybrid Woven Composites via a Novel SHPB-AE Coupled Test. Compos. B Eng..

[18] Martínez-Jequier J., Gallego A., Suárez E., Juanes F.J., Valea Á. (2015). Real-Time Damage Mechanisms Assessment in CFRP Samples via Acoustic Emission Lamb Wave Modal Analysis. Compos. B Eng..

[19] de Groot P.J., Wijnen P.A.M., Janssen R.B.F. (1995). Real-Time Frequency Determination of Acoustic Emission for Different Fracture Mechanisms in Carbon/Epoxy Composites. Compos. Sci. Technol..

[20] Gutenberg B. (1956). The Energy of Earthquakes. Q. J. Geol. Soc..

[21] Rao M.V.M.S., Prasanna Lakshmi K.J. (2005). Analysis of B-Value and Improved b-Value of Acoustic Emissions Accompanying Rock Fracture. Curr. Sci..

[22] Wang J.Y., Chen Z.Z., Wu K. (2019). Properties of Calcium Sulfoaluminate Cement Made Ultra-High Performance Concrete: Tensile Performance, Acoustic Emission Monitoring of Damage Evolution and Microstructure. Constr. Build. Mater..

[23] Scholz C.H. (2015). On the Stress Dependence of the Earthquake b Value. Geophys. Res. Lett..

[24] Shiotani T. (2006). Evaluation of Long-Term Stability for Rock Slope by Means of Acoustic Emission Technique. Ndt E Int..

[25] Shiotani T., Luo X., Haya H., Ohtsu M. Damage Quantification for Concrete Structures by Improved B-Value Analysis of AE. Proceedings of the 11th International Conference on Fracture (ICF11).

[26] Ohno K., Ohtsu M. (2010). Crack Classification in Concrete Based on Acoustic Emission. Constr. Build. Mater..

[27] Sagasta F., Benavent-Climent A., Fernández-Quirante T., Gallego A. (2014). Modified Gutenberg–Richter Coefficient for Damage Evaluation in Reinforced Concrete Structures Subjected to Seismic Simulations on a Shaking Table. J. Nondestr. Eval..

[28] Sagasta F., Zitto M.E., Piotrkowski R., Benavent-Climent A., Suarez E., Gallego A. (2018). Acoustic Emission Energy B-Value for Local Damage Evaluation in Reinforced Concrete Structures Subjected to Seismic Loadings. Mech. Syst. Signal Process..

[29] Mujica L.E., Vehí J., Staszewski W., Worden K. (2008). Impact Damage Detection in Aircraft Composites Using Knowledge-Based Reasoning. Struct. Health Monit..

[30] Talreja R., Phan N. (2019). Assessment of Damage Tolerance Approaches for Composite Aircraft with Focus on Barely Visible Impact Damage. Compos. Struct..

[31] Baker A., Rajic N., Davis C. (2009). Towards a Practical Structural Health Monitoring Technology for Patched Cracks in Aircraft Structure. Compos. Part A Appl. Sci. Manuf..

[32] Ghabezi P., Farahani M., Shahmirzaloo A., Ghorbani H., Harrison N.M. (2020). Defect Evaluation of the Honeycomb Structures Formed during the Drilling Process. Int. J. Damage Mech..

[33] Smith B., Banerjee B. (2012). Reliability of Inserts in Sandwich Composite Panels. Compos. Struct..

[34] Jung D., Yu W.-R., Na W. (2021). Investigation of I B-Values for Determining Fracture Modes in Fiber-Reinforced Composite Materials by Acoustic Emission. Materials.

[35] Jung D., Yu W.-R., Na W. (2020). Use of Acoustic Emission b (Ib)-Values to Quantify Damage in Composites. Compos. Commun..

[36] Jung D., Yu W.-R., Ahn H., Na W. (2022). New B-Value Parameter for Quantitatively Monitoring the Structural Health of Carbon Fiber-Reinforced Composites. Mech. Syst. Signal Process..

[37] (2017). Standard Test Method for Tensile Properties of Polymer Matrix Composite Materials.

